# Pharmacokinetic prediction of an antibody in mice based on an in vitro cell-based approach using target receptor-expressing cells

**DOI:** 10.1038/s41598-020-73255-1

**Published:** 2020-10-01

**Authors:** Yuki Noguchi, Kazuhisa Ozeki, Hidetaka Akita

**Affiliations:** 1 Research Division, Chugai Pharmaceutical Co., Ltd., 1-135, Komakado, Gotemba, Shizuoka 412-8513 Japan; 2 Laboratory of DDS Design and Drug Disposition, Graduate School of Pharmaceutical Sciences, Chiba University, 1-8-1 Inohana, Chuo-ku, Chiba, Chiba 260-0856 Japan

**Keywords:** Drug discovery, Pharmaceutics

## Abstract

In vivo pharmacokinetics (PK) studies using mice and monkeys are the main approaches for evaluating and predicting the PK of antibodies, and there is a strong demand for methods that do not require animal experiments. In this work, we focused on quantitatively predicting the nonlinear PK of an antibody based on cell-based assays. An anti-mouse Fc gamma receptor IIB antibody was used as a model antibody. To determine the PK parameters related to nonspecific elimination in vivo, the plasma concentration profile at 100 mg/kg, at which target-specific clearance is saturated, was analyzed by a 2-compartment model. To estimate the parameters related to target-specific elimination, the Michaelis–Menten constant (K_m_) and the maximum elimination rate (V_max_) were determined by an uptake assay using Chinese hamster ovary (CHO) cells expressing the target receptor. Finally, the integration of all of these parameters permitted the PK to be predicted at doses ranging from 1 to 100 mg/kg regardless of whether target-specific clearance was saturated or nonsaturated. The findings presented herein show that in vitro assays using target-expressing cells are useful tools for obtaining PK parameters and predicting PK profiles and, in some cases, eliminate the need for in vivo PK studies using experimental animals.

## Introduction

Establishing a method for evaluating and predicting antibody pharmacokinetics (PK) that also takes animal welfare into account is an important part of the discovery and development of antibody therapeutics. Multiple processes, i.e., nonspecific binding and pinocytosis, are involved in the elimination of an antibody, recycling via the neonatal Fc receptor (FcRn), and targeting-mediated binding and/or internalization. Because both target-mediated and nonspecific processes are involved in the process of eliminating an antibody from the blood circulation, it is extremely challenging to predict the PK of the antibody^1,2^. Therefore, numerous in vivo studies and in vitro assays have been performed to evaluate PK in animals and predict it in humans^3^.

In vivo-based methods are the most common approaches for evaluating and/or predicting the PK of an antibody. Human FcRn transgenic mice and cynomolgus monkeys are frequently used to reveal the PK of candidate antibodies in vivo, and it is widely accepted that empirical approaches, such as simple allometric scaling, can reliably predict the human linear PK of an antibody that does not show target-dependent elimination^4,5^. However, this approach involves the sacrifice of many experimental animals. A wide variety of in vitro assays have been used to evaluate nonspecific binding and antibody uptake, but none of these assays are able to quantitatively predict PK profiles^6–8^. In addition, cell-based assays such as FcRn-mediated transcytosis assays have been developed to predict the half-life of an antibody, but they can only be used to rank candidate antibodies by half-life^9–12^. For these reasons, an in vivo-based approach would be the most efficient strategy for evaluating the PK of an antibody not cleared by target-dependent elimination in animals and predicting it in humans.

Numerous animals are required to evaluate the nonlinear PK parameters, such as the Michaelis–Menten constant, K_m_, and the maximum elimination velocity, V_max_, of an antibody that is cleared through a target-dependent decay process. In a PK study performed to determine nonlinear PK parameters, Dong et al. reported that 9–18 monkeys were sacrificed^13^. Thus, in vivo-based screening and prediction methods used to evaluate the PK of candidate antibodies are limited in capacity, throughput, and animal welfare considerations. It is particularly noteworthy that numerous animal studies are frequently required to evaluate PK at various dosages to determine PK parameters. It is therefore clear that alternative methods to in vivo PK studies are needed.

Since binding affinity and specificity are key parameters for predicting PK, pharmacological activity and adverse effects, parameters related to binding to a target molecule are generally determined in the initial phase of drug discovery. However, to date, there have been only a few reports regarding the prediction of PK profiles based on parameters determined by in vitro binding studies^3,14^. Surface plasmon resonance spectroscopy using Biacore is widely accepted as an indispensable tool for determining the affinity of an antibody for a target molecule^3,15,16^. However, in some cases, humans exhibit an unexpected PK that is quite different from that observed in mice and monkeys. For example, an anti-Neuropilin-1 antibody was found to be eliminated faster in humans than monkeys even though its binding affinities for mouse, monkey, and human antigens were similar^6,17^. Denosumab is also cleared from the body much faster in monkeys than in humans, even though its affinity is comparable between these species^18^. Therefore, the measurement of binding affinity alone is not sufficient to quantitatively predict PK. One of the limitations of the Biacore system is that it is only able to evaluate binding affinity parameters and cannot be used to estimate cellular binding and uptake. It has been reported that some cell-based approaches, in addition to having the ability to measure affinity, can be used to evaluate target-mediated internalization, and attempts have been made to use such approaches to predict PK profiles. However, in some reports, in vitro kinetic parameters were not quantitatively determined^19,20^.

**Figure 1:**
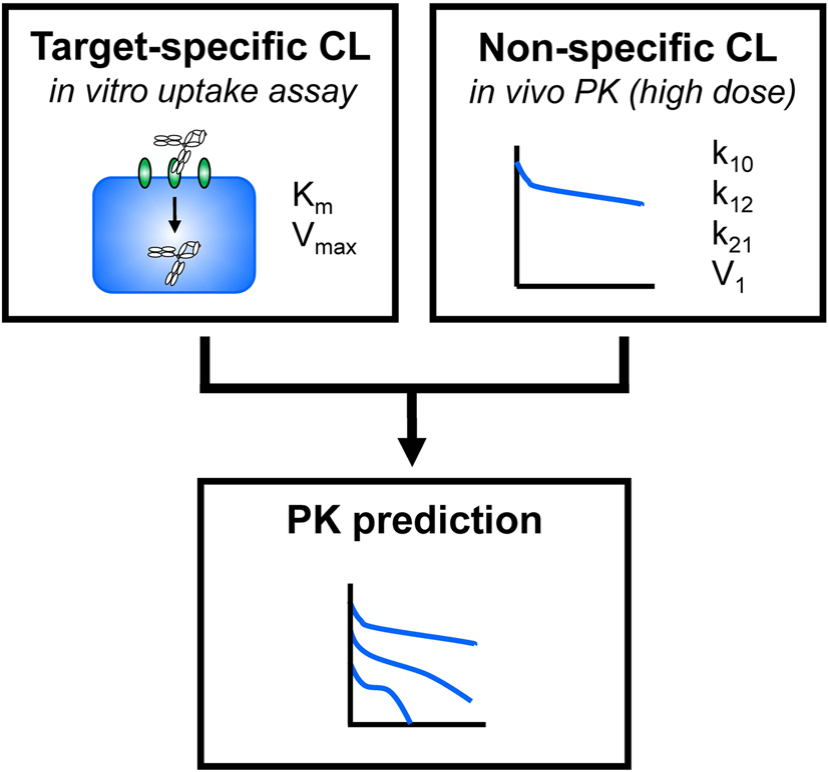
Schematic chart for predicting nonlinear PK using in vivo and in vitro parameters. The diagram outlines the process for predicting the nonlinear pharmacokinetics of the antibody. PK parameters related to nonspecific linear elimination were obtained from an in vivo mouse PK study, and those for describing target-dependent nonlinear elimination were obtained from an in vitro cell-based assay. These parameters were integrated in the 2-compartment model with the Michaelis–Menten equation, and the PK of the antibody was predicted. *CL* clearance.

Here, we report a method that allows the PK of an antibody to be predicted based on in vitro cell-based assays, greatly reducing the number of experimental animals required. We used an antibody against mouse Fc gamma receptor IIB (FcγRIIB)^21,22^, which is expressed mainly in liver sinusoidal endothelial cells and is critically involved in the uptake of an antibody-antigen complex from the circulation^23^, as a model antibody. PK parameters related to nonspecific elimination, i.e., k_10_, k_12_, k_21_, and V_1_ were determined in an in vivo PK study at only a high dose (100 mg/kg), and parameters related to target-mediated elimination, i.e., K_m_ and V_max_, were estimated by in vitro uptake assays using FcγRIIB-expressing Chinese hamster ovary (CHO) cells (Fig. 1).

## Results

### PK of an antibody after intravenous administration in mice

**Figure 2:**
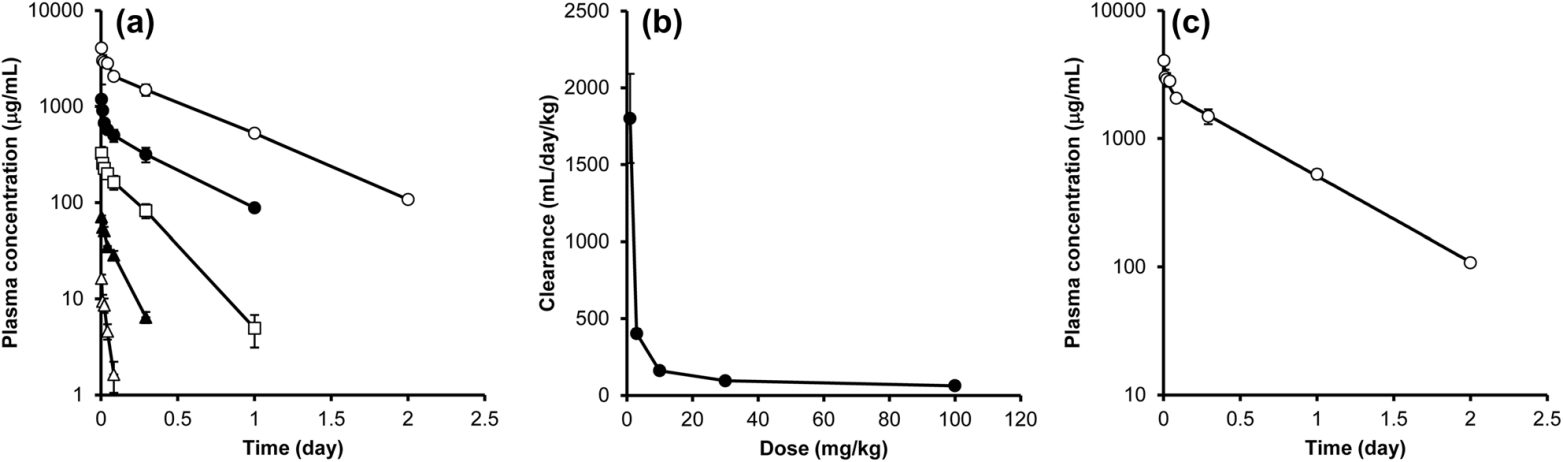
In vivo PK profiles of the antibody in mice. (**a**) Plasma concentration–time profile of the antibody. The antibody was injected into the tail vein of mice at doses of 1 mg/kg (white triangle), 3 mg/kg (black triangle), 10 mg/kg (white square), 30 mg/kg (black circle), and 100 mg/kg (white circle), and blood was collected continuously. The plasma concentrations of the antibody were measured by a ligand binding assay using electrochemiluminescence (ECL). (**b**) The relationship between dose and clearance. Clearance was calculated by noncompartment model (moment) analysis and plotted against the administered dose. (**c**) Fitted curve of the PK profile at 100 mg/kg. Fitting with the 2-compartment model is shown as a solid line. Each vertical bar represents the mean ± SD (n = 3).

To evaluate PK profiles, the antibody was administered to mice at doses ranging from 1 to 100 mg/kg, and the plasma concentration–time profile of the antibody was determined (Fig. 2a). At a high dose (100 mg/kg), the antibody was eliminated with a half-life of approximately 11 h (Supplemental Table S1). In contrast, at a lower dose, the clearance of the antibody was accelerated, and a nonlinear relationship between clearance and dose was revealed. Figure 2b shows the relationship between the administered dose and clearance. Clearance was drastically slower at doses approaching 100 mg/kg than at the minimum dose (1 mg/kg) and reached a plateau at 100 mg/kg. This suggests that target-specific clearance was saturated at 100 mg/kg and that at a higher dose, clearance was largely explained by nonspecific decay. Since clearance at doses of 30 and 100 mg/kg were comparable, the nonspecific clearance mechanism was not saturated at the high dose. To evaluate PK parameters explaining this nonspecific elimination, the PK profile of the antibody at 100 mg/kg was analyzed by means of a 2-compartment model (Fig. 2c), and k_10_, k_12_, k_21_, and V_1_ were estimated as shown in Table 1.

**Table 1:** PK parameters of 100 mg/kg antibody in mice determined by the 2-compartment model.

Parameter	Unit	Mean	CV%
V_1_	mL/kg	25.7	11.3
k_10_	1/day	2.46	11.4
k_12_	1/day	14.2	74.1
k_21_	1/day	25.3	57.5

The mean plasma concentration–time profile of the antibody at 100 mg/kg was analyzed by the 2-compartment model, and the target-independent PK parameters k_10_, k_12_, k_21_, and V_1_ were estimated.

### In vitro cellular uptake assay using mouse FcγRIIB-expressing CHO cells

To quantitatively determine the PK parameters related to target-specific elimination, in an in vitro cell-based assay, the specific assay conditions were investigated. It was confirmed that washing with an acidic buffer completely removed the cell-bound antibody (Supplementary Fig. S1). Thus, it is plausible that the use of an acid wash would permit internalized antibodies to be distinguished from the cell-surface-bound antibodies. Furthermore, a linear increase in uptake was observed for periods of up to 20 min after incubation, as shown in Supplementary Fig. S2.

**Figure 3:**
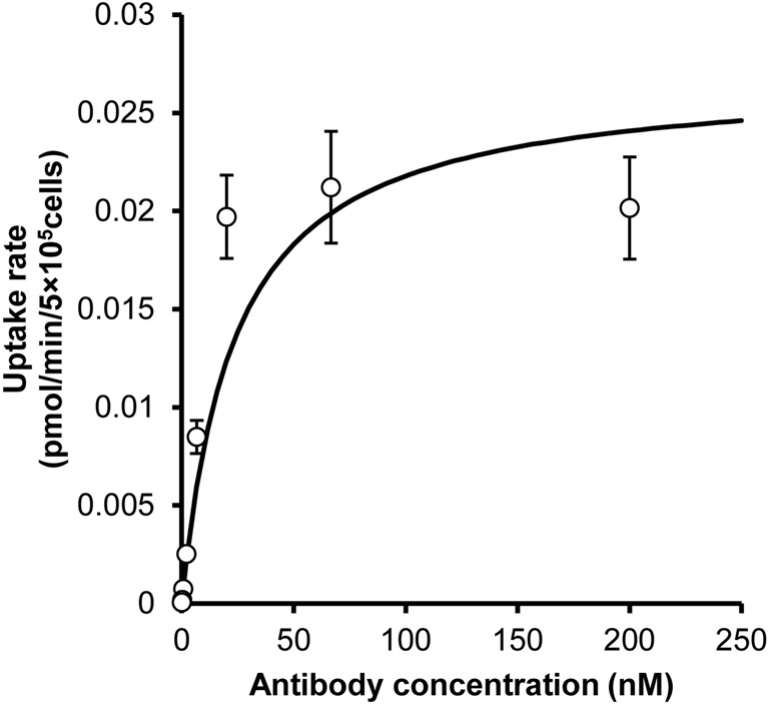
In vitro cell-based uptake assay using FcγRIIB-expressing CHO cells. A ^125^I-labeled antibody was incubated with mouse FcγRIIB-expressing CHO cells at 37° for 15 min, and the amount of internalized antibody was measured after washing with glycine buffer. Uptake velocity was plotted against concentration. The data were fitted to the Michaelis–Menten equation to estimate K_m_ and V_max_. Each point represents the mean ± SD (n = 3).

To determine the PK parameters related to target-specific elimination, the in vitro cellular internalization at 15 min was measured, and the uptake velocity was plotted (Fig. 3). Uptake increased in a concentration-dependent manner and reached a plateau at a concentration of approximately 20 nM. These data were fitted to the Michaelis–Menten equation (Fig. 3). The estimated K_m_ and V_max_ were determined to be 23.6 nM and 0.0269 pmol/min/5 × 10^5^ cells, respectively (Table 2). As shown in Supplementary Fig. S4, analysis with the Hill equation was also performed; however, the obtained parameters were comparable to the values obtained by normal Michaelis–Menten-based fitting.

**Table 2:** In vitro cellular uptake parameters of the antibody in FcγRIIB-CHO cells.

Parameter	Unit	Mean	CV (%)
K_m_	nM	23.6	24.4
V_max_	pmol/min/5 × 10^5^ cells	0.0269	19.2

The concentration-uptake velocity profile of the antibody was analyzed by the Michaelis–Menten equation, and the target-dependent PK parameters Michaelis–Menten constant (K_m_) and maximum elimination rate (V_max_) were estimated.

### In vivo PK prediction using PK parameters obtained from in vivo and in vitro assays

**Figure 4:**
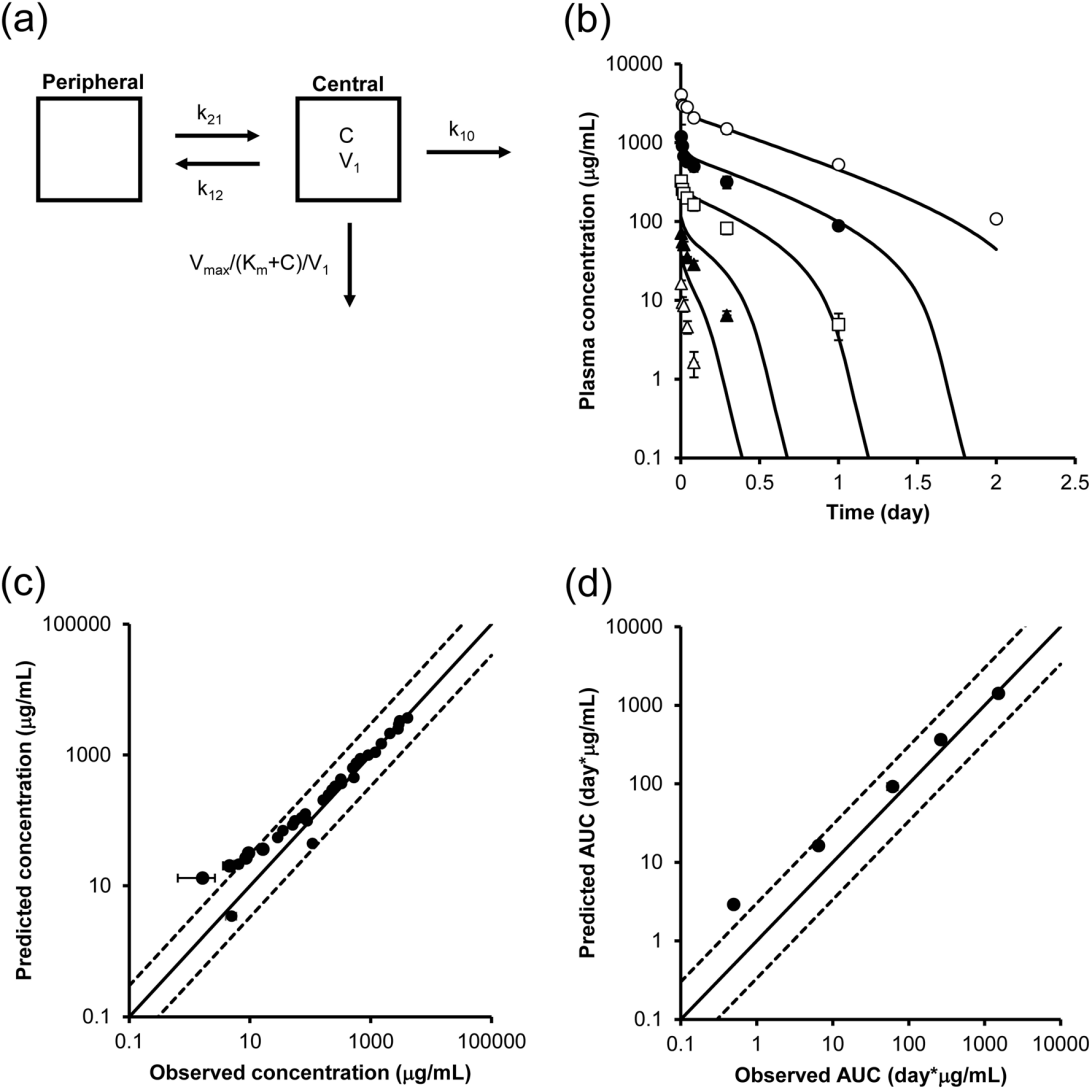
PK prediction using kinetic parameters obtained from in vivo and in vitro cell-based assays. (**a**) PK model for simulating concentration–time profiles of the antibody. A 2-compartment model with the Michaelis–Menten equation was used for PK prediction. The in vitro V_max_ was scaled by the number of target-expressing cells per whole liver, and the velocity per body weight was then calculated using the body weight of the mice. C: concentrations of the antibody. (**b**) Predicted and observed concentration–time profile of the antibody in mice at doses of 1 mg/kg (white triangle), 3 mg/kg (black triangle), 10 mg/kg (white square), 30 mg/kg (black circle), and 100 mg/kg (white circle). The solid line shows the simulated PK profile using in vitro parameters, and the dots show the observed concentrations. Each point represents the mean ± SD (n = 3). (**c**) The relationship between predicted and observed concentrations. (**d**) The relationship between predicted and observed AUC_0–inf_. The solid lines show the lines of unity, and the area between the dotted lines represents an area within a threefold difference.

The PK profile of the antibody was simulated using in vivo- and in vitro-derived parameters to verify the predictability of our approach. As shown in Fig. 4a, a 2-compartment model in combination with the Michaelis–Menten equation was used to describe the PK profile at a dose of 100 mg/kg or less. The V_max_ value determined in vitro (Table 2) was compensated for by the number of target (FcγRIIB)-expressing cells in vivo (i.e., liver sinusoidal endothelial cells and Kupffer cells) based on the assumption that the whole liver contained a total of 7.6 × 10^6^ cells^24^, and the velocity per body weight was then calculated using the body weight of the mice.

Simulated PK profiles at the concentrations observed in mice were determined, and the results are shown in Fig. 4b. Additionally, Fig. 4c shows the 1-to-1 plot of the predicted and observed concentrations. The solid lines are the lines of unity, and the area between the dotted lines represents the area within a threefold difference. Most of the data was within this well-correlated area, while some of the data obtained at low doses were out of this range. Correlations of area under the curve (AUC) values calculated by noncompartment (moment) analysis using observed and simulated PK profiles are also shown in Fig. 4d. In summary, the PK profile of the antibody could be reasonably accurately predicted by integrating PK parameters obtained from an in vivo PK study of only one dose and in vitro cell-based assays.

## Discussion

The purpose of this study was to develop a strategy that can be used to reduce the numbers of animals needed to evaluate and/or predict the PK profile of an antibody by determining parameters related to target-dependent cellular uptake in vitro. To measure parameters related to target-mediated clearance (K_m_ and V_max_), in vitro uptake assays were performed, and the data were analyzed by the Michaelis–Menten model. As a minimally required in vivo study, nonspecific parameters (k_10_, k_12_, k_21_, and V_1_) were determined in vivo by 2-compartment model-based fitting against the plasma concentration–time profile of the antibody at a dose of 100 mg/kg, at which target-specific clearance was saturated. These parameters were then integrated to predict PK profiles (Fig. 1). Such a simple model with a small number of parameters is proposed for minimizing the number of animals needed to predict PK in the early stage of drug discovery from the viewpoint of the 3R (replacement, reduction and refinement) principle.

Ultimately, given animal welfare considerations, all PK prediction studies should be performed in vitro. In vitro assays are frequently used to evaluate nonspecific binding by baculovirus particle-based ELISA^6^, extracellular matrix binding assays^8^, and heparin binding assays^7^. While none of these assays are able to quantitatively predict PK, they can be helpful for designing novel biologics to improve PK. In parallel, cell-based assays such as transcytosis assays and recycling assays using FcRn-expressing cells have also been developed to predict the half-life and clearance of an antibody, but they are only able to rank candidate antibodies based on these parameters^9–12^. Therefore, there is currently no alternative to in vivo studies for evaluating nonspecific elimination.

Empirical approaches such as allometric scaling of monkey PK data have good predictability for the PK of an antibody in humans and are generally accepted for conventional antibodies that do not show target-dependent elimination^4,5^. The plasma concentration of the antibody was much higher (> 100 μg/mL) than the in vitro estimated K_m_ value [23.6 nM (3.5 μg/mL)] (Table 2), suggesting that target-specific elimination was saturated. In contrast, the in vivo clearance of the antibody was comparable at doses of 30 mg/kg and 100 mg/kg. Thus, the machinery associated with target-independent decay was unsaturated. Therefore, PK parameters at a high dose were used as target-independent parameters since the concentrations of the antibody were higher than the K_m_ value estimated from the in vitro uptake study, and saturation of target-specific elimination was observed (Fig. 2b).

While good fitting within a threefold concentration range was observed for the in vitro-based approach, as shown in Fig. 4c, predictability at low concentrations can be improved. To determine the cause of this difference between in vitro-based predictions and in vivo-based predictions, the in vivo PK data shown in Fig. 2a were analyzed by the 2-compartment model with the Michaelis–Menten equation. The in vivo K_m_ was determined to be 42.4 nM, and the in vivo V_max_ was determined to be 0.0627 pmol/min/5 × 10^5^ cell. The binding affinity (dissociation constant: KD) of the antibody for mouse FcγRIIB was reported to be 0.29 nM based on a Biacore assay^25^, and the KD was determined to be 3.3 nM and 2.26 nM by a cellular binding assay, as shown in Supplemental Figs. S3 and S5, respectively. Recombinant protein in buffer was used for the Biacore assay, which might explain the difference in binding to the cell in the presence of other proteins and extracellular matrix. Moreover, the in vitro K_m_ was determined to be 23.6 nM (Table 2), which is larger than both the KD determined using the Biacore assay and the cellular KD determined by the binding assay and is most comparable to the in vivo value. These findings suggest that the uptake assay is a useful method for determining K_m_ and predicting PK. In addition, the in vitro V_max_ value (Table 2) was 2.3-fold lower than the in vivo V_max_ value. The possible reasons for this difference in V_max_ are (1) differences in receptor expression levels and (2) differences in the number of receptor-expressing cells in vitro and in vivo. Compensating the influence of these factors could lead to even more accurate predictions of PK.

Finally, our concept for predicting PK can be helpful for reducing the number of animal experiments needed. Nonlinear PK in humans is generally predicted based on PK parameters determined by studies at various doses (three to five groups) using numerous cynomolgus monkeys (nine to fifteen animals)^13^, resulting in animal welfare concerns. Therefore, a method for predicting PK that is aided by in vitro assays and minimizes the number of animal experiments required would be very desirable. Our approach requires an in vivo PK study in only one group of animals (three animals) to determine PK parameters related to nonspecific elimination. Such an approach would theoretically reduce the number of animals needed to evaluate and predict the nonlinear PK of an antibody by 80%.

However, whether our approach is applicable for predicting the in vivo pharmacokinetics of other antibodies against different target proteins is clearly important. In this study, we demonstrated that our approach can be used to predict the PK for just one antibody against mouse FcγRIIB, which is expressed dominantly in the liver. The expression levels and internalization rates of other antibodies targeting other proteins may be different, which could affect the clearance of the antibody. Therefore, to expand the applicability of our method, further studies of additional preclinical and/or clinical therapeutic antibodies against a nonhepatic target is a necessary next step. Additionally, since the present study is focused on predicting PK in mice in the context of the 3R principle, the predictability of human PK should also be verified by using clinical data and in vitro uptake studies using human tissue-derived cells in the future.

In conclusion, we report an in vitro uptake assay in receptor-expressing cells that can be used to quantitatively determine the PK parameters K_m_ and V_max_ and demonstrate its use in predicting nonlinear PK profiles in mice using in vitro parameters by using an anti-mouse FcγRIIB antibody as a model. Our approach contributes to reducing the numbers of experimental animals needed to evaluate and predict the PK of an antibody.

## Materials and methods

### Reagents

The following materials were purchased from commercial sources: MULTI-ARRAY 96-well SECTOR plates (Meso Scale Discovery, L15XA-3), an anti-human capture antibody (Bethyl Laboratories, A80-319A), a detection antibody (Southern Biotech, #9040-08), SULFO-Tag streptavidin (Meso Scale Discovery, R32AD-1), bovine serum albumin (BSA) fraction V (Roche, 10738328103), and 96-well plates for the cell assay (Sumitomo Bakelite, MS-3296U). Other reagents were purchased from local commercial sources.

### Animals

C57BL/6J mice (6 weeks old, male) were purchased from Charles River Laboratories (Japan).

### Animal experiments

The animal experiments in this study were performed in accordance with the Guidelines for the Care and Use of Laboratory Animals at Chugai Pharmaceutical Co., Ltd., which is accredited by the Association for Assessment and Accreditation of Laboratory Animal Care (AAALAC) International.

### PK study of the anti-mouse FcγRIIB antibody in mice

The PK of the anti-mouse FcγRIIB antibody in plasma was evaluated by administering the antibody at doses of 1, 3, 10, 30, and 100 mg/kg to mice via the tail vein. The drug solution consisted of antibody in phosphate-buffered saline containing Tween 80 and was administered at a volume of 10 mL/kg. Five minutes, 15 min, 30 min, 1 h, 2 h, 7 h, 1 day, and 2 days after injection, blood was collected from the cervical vein without anesthesia and mixed with heparin sodium. Plasma was obtained by centrifuging the blood (15,000 rpm, 4 °C, 10 min).

### Measurement of the antibody in plasma samples by an electrochemiluminescence immunoassay

The antibody concentrations in plasma samples were measured by a sandwich ligand binding assay using electrochemiluminescence (ECL) (Meso Scale Discovery). An anti-human IgG heavy and light chain antibody (Bethyl Laboratories, A80-319A) was added a MULTI-ARRAY 96-well SECTOR plate (Meso Scale Discovery, L15XA-3) at a concentration of 0.5 μg/mL in carbonate-bicarbonate buffer (Sigma-Aldrich, C3041). After incubation for 1 h at room temperature, the solution was removed, and the plate was washed with 1% BSA-PBST, and blocked with 5% BSA-PBST. Diluted plasma samples were applied, and then a biotin-labeled anti-human IgG Fc antibody (Southern Biotech, 9040-08) was added. SULFO-Tag-labeled streptavidin was added, and finally read buffer was applied. The signal was detected with a SECTOR Imager 2400 instrument (Meso Scale Discovery).

### Labeling of the antibody with ^125^I

The antibody was labeled with ^125^I by the IODO-GEN method (PerkinElmer, NEX244). Briefly, the antibody was incubated in an IODO-GEN-coated tube, and the reaction solution was purified to remove free iodine.

### In vitro uptake assay using mouse FcγRIIB-expressing CHO cells

Mouse FcγRIIB-CHO cells were produced by Chiome Bioscience. The labeled antibody was incubated with mouse FcγRIIB-expressing CHO cells (5 × 10^5^/well) at concentrations of 0.01–30 μg/mL for 15 min at 37 °C. After incubation, glycine–HCl buffer (100 mM glycine and 100 mM NaCl, pH 3.0) was added to terminate the reaction. The cells were then washed twice with glycine–HCl buffer to remove the membrane-bound antibodies. To count the number of cells, the cell suspension was transferred to another plate, and a BCA Protein Assay (Thermo Scientific, 23225) was performed according to the manufacturer’s protocol. A 5 N NaOH solution was added to the cells, and the plate was heated for 15 min at 60 °C to dissolve cells. The resulting suspension was analyzed with a Gamma counter (PerkinElmer).

### PK analysis

In vivo PK data were analyzed using Phoenix 64 WinNonlin 6.4 software (ver. 6.4.0.768, Pharsight). A Noncompartment model (moment) analysis was performed to obtain the following parameters: half-life, AUC_0–inf_, clearance, and volume of distribution (V_d_). Two-compartment model analysis was performed to obtain the following parameters: k_10_, k_12_, k_21_, and V_1_. In vitro uptake data were analyzed using SAAMII (Ver. 1.2, Saam Institute). To estimate K_m_ and V_max_, Michaelis–Menten model analysis was performed. For these fitting methods, input data were weighed as the reciprocal of the observed values. A 2-compartment model with the Michaelis–Menten equation was used to describe the PK profile, which was derived from target-independent PK parameters (k_10_, k_12_, k_21_, and V_1_) obtained from the in vivo PK study in mice at a dose of 100 mg/kg and target-dependent PK parameters (K_m_ and V_max_) obtained from the in vitro uptake assay. The in vitro V_max_ value (pmol/min/5 × 10^5^ cells) was converted to the in vivo value by compensating for the number of cells, including liver sinusoidal endothelial cells and liver Kupffer cells (7.6 × 10^6^ cells), which are the main cells that express FcγRIIB in the body^23,24^, and the velocity per body weight was then calculated using the body weight of the mice.

## Supplementary information

Supplementary Information.

## Data Availability

The datasets generated in this study are available from the corresponding author upon reasonable request.
