# Supplementary material for: Pharmacokinetic prediction of an antibody in mice based on an in vitro cell-based approach using target receptor-expressing cells
Source: Sci Rep. 2020 Oct 1;10:16268. doi: 10.1038/s41598-020-73255-1 (PMC7529773; doi:10.1038/s41598-020-73255-1)
Supplement: Supplementary file 1 — Supplementary Information. [file 41598_2020_73255_MOESM1_ESM.docx]

**Pharmacokinetic prediction of an antibody in mice based on an *in vitro* cell-based approach using target receptor-expressing cells**

Yuki Noguchi^1^, Kazuhisa Ozeki^1*^, Hidetaka Akita^2^

**Supplementary Table S1 PK parameters calculated by the non-compartment model (moment) analysis**

| **Dose** | **Half-life (day)** | | **AUC_0-inf_ (μg*day/mL)** | | **Clearance (mL/day/kg)** | | **V_d_ (mL/kg)** | |
| --- | --- | --- | --- | --- | --- | --- | --- | --- |
|  | Mean | SD | Mean | SD | Mean | SD | Mean | SD |
| **1 mg/kg** | 0.027 | 0.005 | 0.498 | 0.0517 | 1801 | 291 | 65.7 | 2.66 |
| **3 mg/kg** | 0.100 | 0.010 | 6.54 | 0.403 | 403 | 29.4 | 55.3 | 3.69 |
| **10 mg/kg** | 0.175 | 0.017 | 61.7 | 9.37 | 161 | 26.2 | 41.0 | 3.67 |
| **30 mg/kg** | 0.381 | 0.044 | 265 | 27.8 | 96.0 | 7.83 | 50.7 | 7.37 |
| **100 mg/kg** | 0.442 | 0.019 | 1517 | 96.1 | 63.2 | 3.67 | 40.0 | 2.80 |

The mean plasma concentration–time profiles of the anti-mouse FcγRIIB antibody at doses of 1 to 100 mg/kg were analyzed with the non-compartment model. AUC_0-inf_: area under the curve; V_d_: volume of distribution.

**Supplementary Fig. S1 Washing the cellular bound antibody with an acidic buffer.**

After the antibody was incubated at concentrations of 0.1 to 100 μg/mL at 4 degrees for 60 min, the cells were washed with an acidic Glycine buffer (pH3.0) once or 3 times to remove cellular bound antibody. Residual ratio against the PBS-wash is shown.

**Supplementary Fig. S2 Time-course uptake assay of the antibody using mouse FcγRIIB-expressing CHO cell.**

The antibody was incubated with mouse FcγRIIB-expressing CHO cells at 37 degrees for a maximum of 60 min, and after washing, the total amount (black circle), the bound amount (white triangle), and the internalized amount (black square) are shown, respectively.

**Supplementary Fig. S3 binding of the antibody in mouse FcγRIIB-expressing CHO cell.**

^125^I-labeled antibody was incubated with mouse FcγRIIB-expressing CHO cells at 4 degrees for 60 min, and the amount of bound antibody was measured. Binding amount was plotted against concentrations. Data were fitted with the receptor-ligand binding equation to estimate KD and maximum binding amount. Each point represents mean ± SD (n=3).

**Supplementary Fig. S4 Fitting analysis of cellular uptake with the Hill equation**

The obtained result for cellular uptake (Figure 3) was analyzed with the Hill equation, $V=\frac{V_{max}*C^{n}}{{K_{m}}^{n}+C^{n}}$, where V_max_ is maximum velocity, K_m_ is Michaelis-Menten constant, C is antibody concentration, and n is the Hill constant. The K_m_, V_max_ and n value was determined as 11.4 nM, 0.0234 pmol/min/5×10^5^cells and 1.17, respectively.

**Supplementary Fig. S5 Fitting analysis of cellular binding with the Hill equation**

The relationship between the antibody concentration and cellular binding (Supplementary Figure S3) was analyzed with the Hill equation, $Binding=\frac{B_{max}*C^{n}}{{KD}^{n}+C^{n}}$, where B_max_ is maximum binding, KD is dissociation constant, C is antibody concentration, and n is the Hill constant. The KD, B_max_ and n value was determined as 2.26 nM, 0.272 pmol/min/1×10^5^cells and 1.18, respectively.
